# Comparative study of dental cephalometric patterns of Japanese-Brazilian, Caucasian and Mongoloid patients

**DOI:** 10.1590/2176-9451.19.4.050-057.oar

**Published:** 2014

**Authors:** Renata Sathler, Arnaldo Pinzan, Thais Maria Freire Fernandes, Renato Rodrigues de Almeida, José Fernando Castanha Henriques

**Affiliations:** 1 PhD in Orthodontics, School of Dentistry —USP/ Bauru.; 2 Professor, Department of Orthodontics, University of Northern Paraná (UNOPAR).; 3 Postdoc in Dentistry, School of Dentistry —USP/ Bauru.

**Keywords:** Ethnic group, Reference standards, Orthodontics

## Abstract

**Introduction:**

The objective of this study was to identify the patterns of dental variables of adolescent Japanese-Brazilian descents with normal occlusion, and also to compare them with a similar Caucasian and Mongoloid sample.

**Methods:**

Lateral cephalometric radiographs were used to compare the groups: Caucasian (n = 40), Japanese-Brazilian (n = 32) and Mongoloid (n = 33). The statistical tests used were one-way ANOVA and ANCOVA. The cephalometric measurements used followed the analyses of Steiner, Tweed and McNamara Jr.

**Results:**

Statistical differences (P < 0.05) indicated a smaller interincisal angle and overbite for the Japanese-Brazilian sample, when compared to the Caucasian sample, although with similar values to the Mongoloid group.

**Conclusion:**

The dental patterns found for the Japanese-Brazilian descents were, in general, more similar to those of the Mongoloid sample.

## Abstract (pt)

**Objetivo:**

as características cefalométricas dentárias variam consideravelmente entre as diferentes raças. No Brasil, em virtude da grande miscigenação populacional, é necessário conhecer, também, as variações apresentadas pelas misturas dessas raças. Por essa razão, o objetivo desse estudo foi identificar o padrão das variáveis dentárias de jovens mestiços nipo-brasileiros com oclusão normal e compará-los com amostras semelhantes de leucodermas e de xantodermas.

**Métodos:**

foram utilizadas 40 telerradiografias de jovens leucodermas, 32 de nipo-brasileiros e 33 de xantodermas. As rês amostras apresentavam indivíduos com oclusão normal e face bem balanceada. Foram realizadas análises estatísticas de variância a um critério (ANOVA) e a de covariância (ANCOVA). Basicamente, as variáveis cefalométricas usadas seguiram as preconizadas por Steiner, Tweed e McNamara Jr.

**Resultados:**

encontrou-se diferença estatística (p < 0,05) entre as raças em cinco das variáveis estudadas. Essas diferenças indicaram menor ângulo interincisivos e menor sobremordida para a amostra nipo-brasileira, com relação à amostra leucoderma, mas com valores semelhantes aos da amostra xantoderma.

**Conclusão:**

os valores dentários encontrados para os jovens nipo-brasileiros foram, em geral, mais similares àqueles da amostra de xantoderma.

## INTRODUCTION

In 1899, when the study of malocclusion was limited to dental casts, Angle proposed his classification.^2^ Since then, several authors have dedicated to improve the diagnosis methodology used at that time.^1^

The creation of cephalostat enabled us to study the alterations that occur in the face and cranium, which, in turn, allowed us to make better diagnoses and conduct more accurate treatment. As a consequence, it became necessary to determine standard variables to guide interpretation of results. Additionally, it also became interesting to study the mean cephalometric values of groups with normal occlusion and satisfactory skeletal pattern. Therefore, many analysis methods were created and Downs,^6^ Tweed,^27^ Steiner^24^ as well as many others explored the development of standards that would give support to the clinical field.

It is known that skeletal and facial structures directly influence the position of upper and lower teeth as well as the appearance of the facial profile. Therefore, the mean cephalometric variables must be explicit to the orthodontic community in order to improve treatment offered to patients, including descendants of different races. This is due to the fact that the relation of normality between skeletal and dental positions may be greatly diverse due to ethnic variations^22,25^. Thus, this was the basis on which this study was conducted.

Keeping those reflections in mind,^6,26^ the objective of this research was to determine the mean dental cephalometric variables of young Japanese-Brazilian descendants with normal occlusion, and compare the results with the values of two other groups: Caucasian and Mongoloid. Although many papers on cephalometry are available in the scientific literature, to date, no research has been conducted on this subject.

## MATERIAL AND METHODS

### Material

The sample comprised 105 lateral cephalometric radiographs of 32 young Japanese-Brazilians, 40 young Caucasian subjects and 33 young Mongoloid, all with normal occlusion and well balanced face. Radiographs were retrieved from the archives of the Department of Orthodontics, School of Dentistry, University of São Paulo/Bauru.

**Figure 1:**
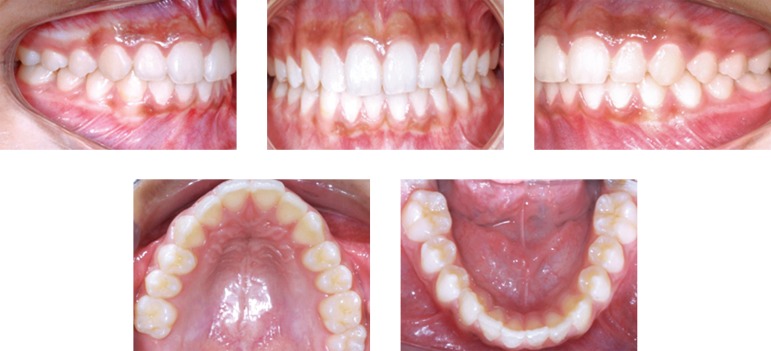
Occlusal characteristics of a female patient from the Japanese-Brazilians sample.

The sample presented with all permanent teeth in occlusion (the presence of second and third molars was optional), absence of orthodontic treatment, normal occlusion or Class I malocclusion. Crowding not greater than 2 mm was acceptable (Fig 1).

Caucasian and the Mongoloid samples should have both parents Caucasian and Mongoloid, respectively. This means that there was an absence of racial miscegenation. On the contrary, the Japanese-Brazilian sample was represented by descents of both Caucasian and Mongoloid.

There was also the concern of selecting a nearly identical number of boys and girls for the samples of Caucasian (20 of each sex), Japanese-Brazilian (17 females and 15 males) and Mongoloid (17 females and 16 males), thus providing homogeneous groups in relation to sex.

### Methods

The three groups were radiographed in maximum intercuspation, since the difference between this position and the mandibular centric relation is minimal at this age and slightly affects the cephalometric results, especially in cases of normal occlusion.^30^

After the anatomical drawing, landmarks were identified and subsequently scanned using Numonics, AccuGrid A30TL (Numonics Corporation, Montgomeryville, PA, USA). The magnification factor was corrected (6% for the Caucasian sample, 9.8% for the Japanese-Brazilian sample and 7% and 8% for the Mongoloid sample) by the software itself.

**Figure 2:**
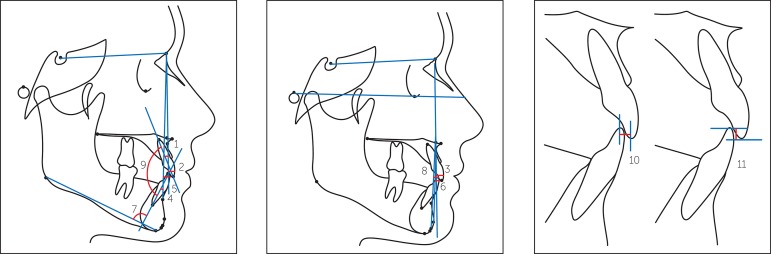
Cephalometric variables.

The variables studied were: 1.NA, 1-NA, 1-Aperp, 1.NB, 1-NB, 1-AP, IMPA, I Line, 1.1, Overjet and Overbite (Fig 2).

### Method error

Tracings and measurements were performed by the same examiner who also transferred data to the software Dentofacial Planner 7.02 (Dentofacial Planner Software Inc., Toronto, Ontario, Canada). After twenty days, 20 radiographs were randomly selected and retraced (both manually and digitally) to determine reliability of results.

Systematic and random errors were evaluated independently for each variable. T-test was used to calculate systematic error,^11^ whereas Dahlberg's formula was used to calculate random error (Table 1).

**Table 1:** Random error (Dahlberg formula) and systematic error (t test)

Variable	1^st^ Tracing	2^nd^ Tracing	Dahlberg	p
	Mean ± SD	Mean ± SD		
1.NA	24.62 ± 4.52	25.16 ± 4.20	1.410	0.518
1-NA	5.32 ± 2.22	5.25 ± 2.14	0.510	0.583
1-Aperp	6.35 ± 1.86	6.18 ± 1.73	0.422	0.240
1.NB	26.36 ± 7.48	26.99 ± 8.09	0.956	0.032*
1-NB	5.24 ± 2.49	5.29 ± 2.46	0.347	0.593
1-AP	3.47 ± 2.32	3.55 ± 2.34	0.208	0.258
IMPA	92.02 ± 7.94	92.52 ± 8.63	0.961	0.219
I Line	-3.99 ± 2.52	-4.24 ± 2.44	0.250	0.031*
1.1	126.95 ± 10.35	125.81 ± 10.22	1.263	0.047*
Overjet	2.82 ± 0.88	2.62 ± 0.72	0.272	0.011*
Overbite	2.22 ± 1.26	2.05 ± 1.14	0.293	0.177

* Significant for P < 0.05.

### Descriptive and comparative analyses

Before proceeding with descriptive and comparative analyses, the variables were submitted to Kolmogorov-Smirnov test which confirmed the normal distribution of the samples and allowed the use of parametric tests.

Due to lack of compatibility between patients' age in the Mongoloid group and to avoid interference in the results, ANCOVA (covariance analysis) test was performed with patients' age considered as the adjustable variable, thereby permitting approximation of values. The results obtained showed that age did not influence any of the studied variables, thus allowing reliable statistic comparison. ANOVA test was used to compare patients' age (Table 2). Results were considered statistically significant for P < 0.05. All aforementioned tests were performed using Statistica for Windows 7.0 software (Statistica for Windows 7.0 Copyright StatSoft. Inc. Tulsa, Okla, USA. http://www.statsoft.com).

**Table 2:** Minimum, maximum and mean age of Caucasian, Japanese-Brazilian and Mongoloid samples. ANOVA followed by Tukey test.

Age	Caucasian		Japanese-Brazilian		Mongoloid	
	Male	Female	Male	Female	Male	Female
Minimum	12.00 ± 12.00		12.97 ± 11.83		11.84 ± 8.35	
Maximum	14.92 ± 14.92		16.62 ± 15.36		21.99 ± 18.46	
Mean	13.57 ± 13.70		14.79 ± 13.22		15.56 ± 15.65	
**ANOVA followed by Tukey test**						
	Caucasian	Japanese-Brazilian		Mongoloid		P
Overall mean	13.64^a^	13.96^a^		15.61^b^		0.000*

* Significant for P < 0.05.

Different letters account for significant differences.

## RESULTS

### Random and systematic error

Table 1 presents the statistical analysis performed to assess intra-examiner error. Systematic errors were found in four variables and random errors were considered acceptable.

### Age means

In order to analyze the minimum, maximum and mean age of Caucasian, Japanese-Brazilian and Mongoloid groups, the samples were separated by sex (Table 2).

### Comparative analysis between groups

ANCOVA was applied to verify potential differences between groups (Table 3).

## DISCUSSION

Due to being practical and of simple comprehension, cephalometry is still frequently used for orthodontic teaching. Undergraduate students with little experience need clear and well-defined parameters to understand the purposes and limitations of orthodontic treatment. With practice, the student gradually gets rid of rigid numbers and goes on to value other subjective aspects such as patient's face, profile and expectations. However, it is impossible to bring forth maturity before establishing the goals and patterns of normality.^12,24^

As for more experienced orthodontists, cephalometric values are the possibility to locate and quantify the problem, which allows them to choose the most appropriate treatment for the patient.

The authors who laid the groundwork of cephalometric analysis developed their norms based on Caucasian samples.^6,16,24,26^ Later on, it was suggested that differences in normality values existed between different racial groups.^5,7,10,18,19^

With these reasons in mind, the need to specify the cephalometric pattern for Japanese-Brazilians (descents of Caucasian Brazilians and Japanese) became evident.

### Method error

For random errors, the limits for the linear variables were set at 1 mm, whereas for the angular variables they were set at 1.5°. Important random errors were not found (Table 1).

Among the 11 studied variables, four presented statistically significant differences between the first and the second measurements (Table 1).

Since the variables were used for comparison purposes,^24^ the application of these values was considered reliable. Additionally, the absence of random errors increases reliability of results.

### The sample

In order to standardize the results, the sample was selected on the basis of patients' lineage and occlusion. This is because gathering a sample in which other variables are equivalent allows us to obtain results derived solely from racial differences between groups. Clearly, the strict inclusion criteria applied to the sample limited its size. However, a choice was made to sacrifice the number of subjects included in the research in order to enhance homogeneity of the sample. Before comparing the groups, a comparison between patients' sex was performed.^7^ Since no statistically significant differences were found between them, both male and female patients were included in all three groups (Table 3).

**Table 3:** Comparison between groups. ANCOVA and Tukey test.

Variable	Caucasian	Japanese-Brazilian	Mongoloid	p value age	p value group
	Mean ± SD	Mean ± SD	Mean ± SD		
**Upper incisor in relation to the maxilla **					
1.NA	21.69 ± 5.50	24.80 ± 5.87	23.14 ± 6.88	0.246	0.074
1-NA	4.08 ± 2.07	4.77 ± 2.04	4.49 ± 2.37	0.661	0.432
1-APerp	5.24 ± 1.53	6.08 ± 1.79	5.56 ± 1.81	0.900	0.122
**Lower incisor in relation to the mandible**					
1.NB	25.95 ± 5.57	27.70 ± 7.39	28.54 ± 4.74	0.273	0.095
1-NB	4.38^a^ ± 1.63	5.29^a.b^ ± 2.29	5.70^b^ ± 1.93	0.647	0.017*
1-AP	2.32^a^ ± 1.80	3.11^a.b^ ± 1.95	3.47^b^ ± 1.80	0.903	0.042*
IMPA	92.50 ± 6.65	93.40 ± 8.53	93.95 ± 6.07	0.369	0.497
I line	-2.83^a^ ± 1.84	-3.82^a.b^ ± 2.17	-4.22^b^ ± 1.85	0.584	0.010*
**Relationship between incisors**					
1.1	130.09^a^ ± 7.96	124.88^b^ ± 9.62	125.39^b^ ± 7.79	0.054	0.004*
Overjet	2.66 ± 0.66	2.96 ± 1.22	2.79 ± 0.76	0.225	0.285
Overbite	2.99a ± 1.15	2.23^b^ ± 1.40	2.57^a.b^ ± 1.11	0.138	0.041*

* Significant for P < 0.05.

Different letters account for significant differences.

The age group of choice was based on Ceylan, Baydas and Bolukbasi's^4^ findings in which the majority of patients submitted to orthodontic treatment is aged between 10 and 14 years old. Therefore, this age group has priority in obtaining standard norms. Additionally, determining a particular age group favors comparisons with past and future studies.

### The choice of variables

The criteria applied in selecting the variables were based on reliability, but also on frequency of their use in orthodontics.^12^ This facilitates a rapid visualization of results and future comparisons with other studies.

Only statistically different variables were discussed after comparison with the Caucasian and Mongoloid groups (Table 3). These variables were gathered according to their representativity so as to facilitate comprehension. Results were discussed focusing on the Japanese-Brazilian sample and their differences to the Caucasian and Mongoloid samples.

### Lower incisors linear alterations (1-NB, 1-AP and I Line)

The Japanese-Brazilian sample showed accentuated protrusion of lower incisors. Their values were in between those of the Caucasian and Mongoloid samples. It is worth noting that all three linear variables concerning the horizontal position of incisors showed the same behavior, thereby indicating statistic similarity between the Japanese-Brazilian and the other samples. Moreover, all variables showed greater protrusion of lower incisors when the Mongoloid were compared to the Caucasian sample.

Miura, Inoue and Suzuki^18^ also observed great protrusion of Japanese lower incisors in comparison to Caucasian. The results found by Uesato et al,^28^ Engel and Spolter,^7^ Miyajima et al,^19^ Raddi^23^ and Takahashi^25^ also demonstrate greater protrusion of lower incisors in Asiatic samples.

Incisors positioning strongly influences the lower third of the face,^16,29^ specially the lower lip.^8,9^

It is important to observe that the values found for Japanese-Brazilian sample were in an intermediary rank in relation to the Mongoloid and Caucasian samples. This fact may be caused by the miscegenation of this group, since these results were similar for all three variables.

### Upper and lower incisors angular relationships (1.1 and Overbite)

These variables were expected to present statistically significant differences, since some dental variables of the Japanese-Brazilian sample were numerically different from the Caucasian and Mongoloid samples (Table 3).

Variable 1.1 (interincisors angle) in the Japanese-Brazilian sample exhibited lower values than in the Caucasian sample and was similar to the Mongoloid sample, thus representing greater vestibular inclination of incisors among Japanese-Brazilian and Mongoloid, which corroborates the findings of Miura, Inoue and Suzuki^18^.

Values found for overbite also suggest vestibular inclination: the Caucasian sample presented greater overbite than Japanese-Brazilian and Mongoloid samples, which is in agreement with Engel and Spolter's^7^ studies. This may be explained by the fact that greater buccal inclination of incisors reduces vertical trespass, which was also observed by Iwasawa, Moro and Nakamura.^14^

Both interincisors angle and overbite are deeply connected to the angular position of upper and lower incisors.^15,20^ Thus, it is possible to affirm that this characteristic was the determining factor for variable 1.1 to present smaller values than the Caucasian sample, thereby reinforcing the understanding of a more vestibular inclination of lower incisors.^15^ Additionally, it is possible to suggest that this vestibular inclination influenced overbite results.^15,20^

### General considerations

The results yielded by orthodontic treatment in contemporary society not only have to establish a functional and balanced occlusion, but also have to provide pleasant facial esthetics.^17^

Therefore, the expectation of producing facial improvements as a result of tooth movement remains evident, since there is an intimate connection between labial posture and subjacent structures such as the teeth and the alveolar process.

Accepting that environmental influences play an important role in orthodontic treatment and its esthetic results is critical to understand the value and necessity of individualized orthodontic treatment as well as the study of specific norms concerning ethnic groups from different backgrounds.

Considering the anthropological differences of facial and dental patterns, Miura, Inoue and Suzuki^18^ compared a Mongoloid sample with the values of Steiner's analysis. The authors concluded that treatment objectives set for Japanese must be different from those set for Caucasians.^18^

Miura, Inoue and Suzuki^18^ assert that results produced by orthodontic treatment performed in Japanese patients will not be more functional, stable or desirable if we use smaller values for the variables ANB, 1.NA, 1-NA, 1.NB and 1-NB, as advocated by Steiner for American patients.

This means that, in spite of the amount of researches addressing cephalometric patterns, the professional must be wise in order to make good use of these norms. Simply knowing so many different patterns and analyses is not sufficient. The orthodontist must have the ability to apply such knowledge to each specific case.^28^

Since the mean values change according to the sample and depend on the locality of the study, we would agree that individualized values are more applicable. After all, different ethnic groups need different cephalometric patterns.

In general, we may say that the most important factor clinically exhibited by young Japanese-Brazilian in this study is the greater buccal inclination and protrusion of incisors. This aspect must be considered when choosing a treatment protocol. Bearing in mind that this ethnic group frequently presents protrusion and crowding, it is necessary to take into account the limitation of these patients in relation to the amount of incisor retraction.

Furthermore, oral muscles are strong enough to cause treatment relapse. Even the position of lips influences incisors stability and alignment.

Therefore, the decisions about the need for extractions and type of mechanics should be made on the basis of these circumstances.^21^ In case of doubt, it is essential to consider that, to this group, it is more adequate to place lower incisors in a more anterior position, which reduces the indication of extractions in borderline cases. In those cases, only interproximal wear would be able to provide dental alignment.

Therefore, in addition to considering patient's needs, the orthodontist should also know the values of normality for Japanese-Brazilian patients in order to choose the best treatment plan. In clinical terms, we can apply the same principles when selecting pre-adjusted brackets for Class I and II malocclusions cases. In other words, it is possible to apply the results of interincisors angle and overbite found in this research in order to choose the most adequate orthodontic accessories. Brackets used to correct Class I and Class II malocclusion exert greater buccal inclination on lower incisors, whereas those used to correct Class III malocclusion exert greater buccal inclination on upper incisors.^3^

Therefore, a combination of these different prescriptions would allow greater upper and lower buccal tipping and, as a result, could offer more satisfactory esthetic outcomes, in addition to increasing case stability.

In short, based on the results of this study, the objectives of orthodontic treatment conducted with Japanese-Brazilian patients (Mongoloid and Caucasian descendent), must be different from those of Caucasian Brazilian patients.

According to the literature, the use of specific and individualized patterns helps to achieve treatment stability^18^ and adequacy of bone, dental and tegumentary structures.^18,19^

Applying these variables to Japanese-Brazilian born in Brazil allows us to provide more stable and esthetic results. Last but not least, the study by Miyajima et al^19^ reveals the rebirth of an ethnic pride, particularly in large urban centers. Furthermore, there is a growing demand for exclusive orthodontic treatment based on patterns directed towards specific ethnic groups.^13^

## CONCLUSIONS

Based on the methodology applied and on the results of this research, it is possible to conclude that the sample of Japanese-Brazilian presents:

Smaller values for interincisors angle and overbite in relation to the Caucasian sample, but close to Mongoloid values.

Young Japanese-Brazilians present, in general, intermediate numerical values that are in between the samples studied. Nevertheless, Japanese-Brazilian are more similar to Mongoloids than to Caucasians.
